# Cell-Type-Tailored Hydrogels for Adoptive Cell Therapy in Cancer

**DOI:** 10.3390/gels12080678

**Published:** 2026-08-01

**Authors:** Jin Hak Shin, Yeonju Song, Jeehun Park

**Affiliations:** 1 Multidimensional Genomics Research Center, Kangwon National University, 1 Kangwondaehak-gil, Chuncheon 24341, Republic of Korea; 2 Department of Materials Science and Engineering, Seoul National University, 1 Gwanak-ro, Seoul 08826, Republic of Korea; 3 Department of Molecular Bioscience, College of Biomedical Science, Kangwon National University, 1 Kangwondaehak-gil, Chuncheon 24341, Republic of Korea

**Keywords:** adoptive cell therapy, CAR-T, CAR-NK cells, gamma-delta T cells, dendritic cells, macrophage engineering, injectable hydrogels, granular hydrogels, immune cell delivery, cancer immunotherapy

## Abstract

Hydrogel-enabled adoptive cell therapy (ACT) offers a localized and controllable strategy for improving cellular immunotherapy in solid tumors. Hydrogels can enhance cell retention and persistence, support immune cell function within the tumor microenvironment, and reduce systemic toxicity associated with broadly delivered immune stimulants. However, delivery of living immune cells imposes practical constraints on hydrogel selection, including cytocompatible encapsulation, minimal handling and injection stress, adequate transport of oxygen and soluble cues, and an appropriate balance between local retention and timely cell egress. This review summarizes natural, synthetic, and hybrid hydrogel platforms and compares physical/supramolecular assembly, covalent and enzymatic crosslinking, and photo-crosslinking. Injectable in situ-forming depots and shear-thinning/self-healing gels are highlighted for locoregional administration. Key design and reporting dimensions of hydrogels are linked to immune cell outcomes relevant to ACT. These properties include mechanics and viscoelasticity, porosity and mass transport, degradability and remodeling, bioadhesion and extracellular matrix (ECM) mimicry, and immunogenicity versus immune shielding. Finally, a cell-type-tailored framework is presented for chimeric antigen receptor T (CAR-T), T cell receptor-engineered T (TCR-T), and tumor-infiltrating lymphocyte (TIL) products, natural killer (NK) cells, and dendritic cells (DCs) or macrophage/monocyte-derived effectors. Distinct biological requirements are used to motivate corresponding material architectures and cue presentation strategies. The review also provides quantitative reporting guidance, identifies evidence gaps for γδ T cells, and discusses in vivo validation, combination ACT strategies, and translational handling constraints.

## 1. Introduction

ACT has fundamentally reshaped the landscape of cancer immunotherapy by enabling the direct use of living immune cells as therapeutic agents [1]. Recent advances in CAR-T cells, TCR-T cells, TILs, and NK cell therapies, including CAR-NK products, highlight the rapid expansion of ACT beyond its initial successes in hematologic malignancies [2]. In particular, CAR-T and TCR-T platforms have demonstrated that cellular therapies can produce profound and durable antitumor responses in selected settings [3]. Nevertheless, despite these achievements, the broader application of ACT remains constrained by several fundamental barriers, especially in solid tumors [4]. Transferred cells often show limited persistence, inadequate infiltration into the tumor core, reduced viability after infusion, and rapid functional decline within the immunosuppressive tumor microenvironment (TME) [5,6]. Systemic administration of potent immune cells or cytokines also cause off-tumor effects and other toxicities, underscoring the need for more localized and controllable delivery strategies [7].

Hydrogels have attracted increasing attention as biomaterial platforms for addressing many of these challenges. Recent studies emphasize their ability to spatially and temporally regulate immune cell delivery, cytokine release, checkpoint modulation, and local immune activation in space and time [8,9]. Because hydrogels can recapitulate selected features of the ECM, they can create supportive niches that improve cell retention, survival, and local function after implantation or injection [10]. Their high water content, tunable mechanics, and compatibility with diverse crosslinking chemistries allow them to act not only as passive carriers but also as active regulators of immune cell behavior. Hydrogels can serve as localized depots for therapeutic cells, cytokines, chemokines, adjuvants, or checkpoint modulators, thereby enabling spatiotemporal control over immune activation and transport [11,12]. Injectable and in situ-forming systems are especially attractive because they can be delivered minimally invasively, conform to irregular tumor beds, and retain therapeutic cargo at the target site. Such localized control may be essential for overcoming the spatial and immunological barriers that limit ACT efficacy in solid tumors [13,14].

At the same time, hydrogel performance is not determined by a single material property. Immune cells respond to the combined effects of matrix stiffness, viscoelasticity, pore structure, degradability, ligand presentation, and local transport of oxygen, nutrients, and signaling molecules [2,15,16]. These design variables can influence whether transferred cells remain quiescent, activate, proliferate, differentiate into functional effectors, or become exhausted. For this reason, hydrogel design must be aligned with the biology of the intended cell product rather than applied as a generic support matrix [17]. A formulation suited to prolonged T-cell activation, for example, may not provide the same benefit to NK cells, DCs, or macrophage-based products, which differ in their activation cues, trafficking behavior, and functional outputs.

This issue is especially important as ACT expands beyond conventional T-cell therapies. NK cells, TILs, DCs, and macrophage- or monocyte-derived effectors are increasingly being explored as therapeutic candidates. Each population has a distinct therapeutic logic and therefore distinct biomaterial requirements. NK cells may require sustained cytokine support and a microenvironment that preserves cytotoxic activity [18]. DCs require support for antigen loading, maturation, and migration to efficiently prime adaptive immunity [19]. Macrophage-based strategies depend on reprogramming polarization and reshaping the TME toward an antitumor state [20]. These differences suggest that the most effective hydrogel platforms will be those designed around cell-type-specific needs rather than around ACT in general.

However, a critical conceptual gap remains. Hydrogel design strategies are often developed without systematically accounting for the distinct biological requirements of different immune cell types [21]. This limitation hinders the rational optimization of biomaterial platforms and may partly explain the inconsistent translational outcomes observed across different ACT modalities. Although recent reviews have discussed hydrogels for immunomodulation and cell delivery, many focus on T-cell applications or on general material principles. Thus, a more integrated, cell-oriented design framework is needed.

**Figure 1:**
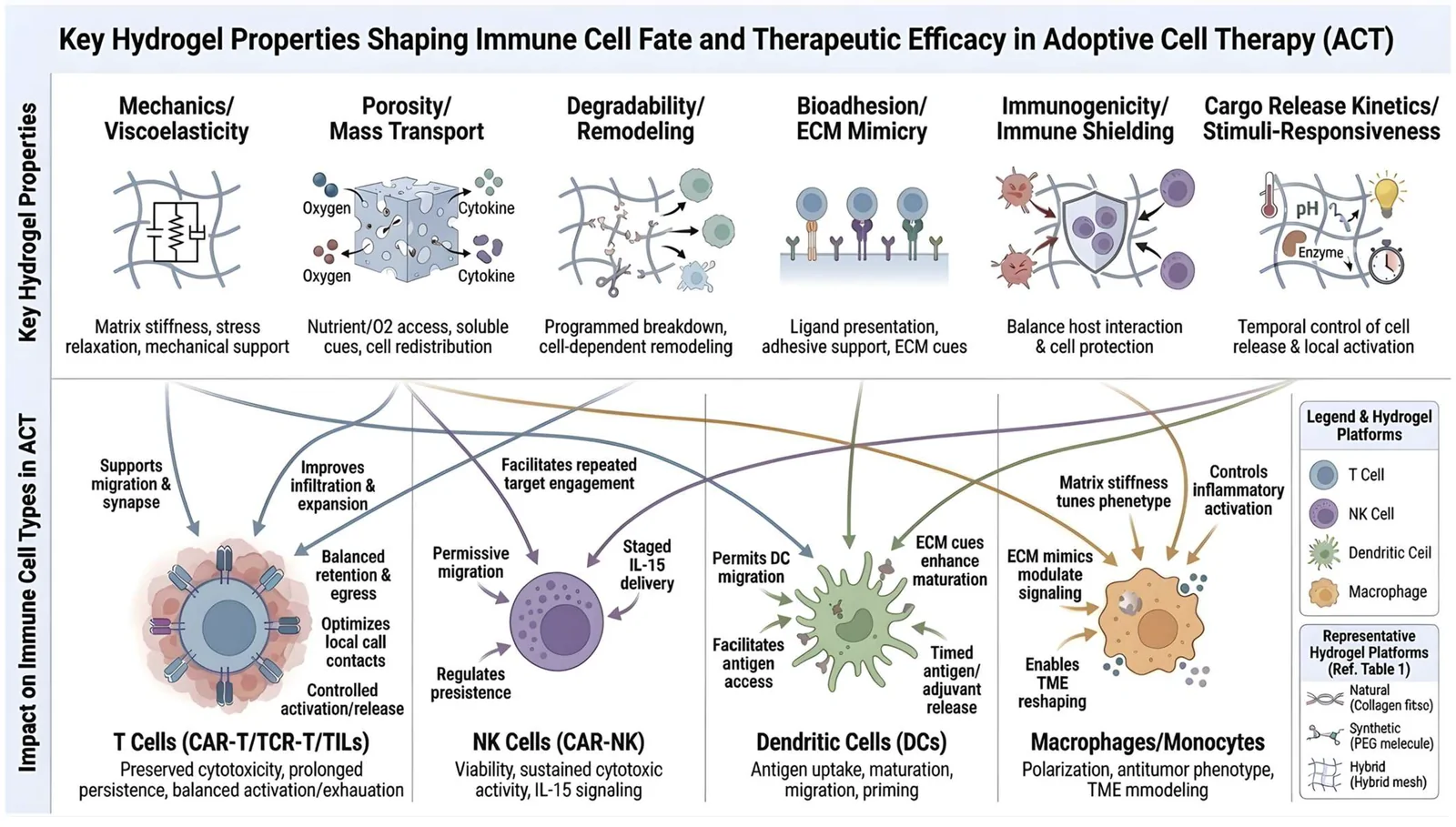
Key hydrogel properties shaping the fate and therapeutic efficacy of adoptively transferred immune cells. The schematic illustrates how mechanics/viscoelasticity, porosity/mass transport, degradability/remodeling, bioadhesion/ECM mimicry, immunogenicity/immune shielding, and stimuli-responsive properties can differentially influence lymphoid and myeloid products used in ACT.

In this review, we focus on cell-type-tailored hydrogel design for ACT. The primary scope is on hydrogel-assisted delivery, retention, and local support of live adoptively transferred immune cells; acellular or hybrid immunomodulatory depots are discussed only when they directly support transferred-cell function or provide mechanistic design principles relevant to cell-containing ACT systems. We first summarize hydrogel platforms, fabrication routes, and cell-compatible preparation strategies for immune cell delivery. We then discuss material properties that govern immune cell fate and provide reporting considerations for mechanics, pore architecture, degradation, swelling, release kinetics, and injection-related parameters. Finally, we examine how hydrogel design can be matched to T-cells, NK cells, γδ T-cells, DCs, and macrophage/monocyte-derived effectors. Throughout, we distinguish evidence-supported principles from emerging hypotheses and discuss in vivo validation, combination ACT, manufacturing constraints, and remaining limitations (Figure 1).

## 2. Hydrogel Platforms, Fabrication, and Formation Strategies for Living Cell Delivery

For ACT, a hydrogel serves as both a delivery depot and a processing environment for living immune cells. During loading, injection, and residence after administration, therapeutic cells encounter precursor chemistry, osmotic and mechanical stress, shear exposure, oxygen and nutrient gradients, and physical constraints on migration or release. These conditions distinguish ACT depots from conventional drug-delivery gels. The central design question is not which hydrogel class is intrinsically superior, but which platform best preserves cell competence while balancing injectability/in situ gelation, local retention, cue presentation, and timely cell release [9,17,22].

### 2.1. Natural, Synthetic, and Hybrid Platforms

Hydrogels for living immune cell delivery are commonly classified as natural, synthetic, or hybrid [23]. This distinction captures a central design trade-off between biological familiarity and engineering control. Natural polymers, including collagen, fibrin, hyaluronic acid, alginate, chitosan, gelatin, and decellularized ECM-derived matrices, can support mild encapsulation, provide ECM-like cues, and permit short-term survival or migration [22,24,25]. Their main limitations include source variability, impurity profiles, batch-to-batch differences, limited independent tunability, and sterilization challenges, all of which are important for products manufactured under Good Manufacturing Practice (GMP) conditions [22,24]. Natural matrices may be valuable when matrix-derived adhesion, remodeling, or innate immune sensing is intentional, but they should not be assumed to be universally superior for ACT.

Synthetic hydrogels, including polyethylene glycol (PEG)-based and other engineered networks, provide greater control over precursor purity, mechanics, degradation logic, ligand density, and release kinetics [9,26,27]. Their main limitation is biological inertness unless they are functionalized. Without programmed adhesive ligands, degradable linkers, cytokines, or immunostimulatory cues, synthetic networks may retain cells without supporting activation, migration, or productive cell–cell interactions. Their principal advantage for ACT is modularity: injectability, mechanics, cue presentation, and degradation can be tuned separately rather than inherited from the base material [9,28].

Hybrid hydrogels combine these strengths and are best viewed as modular architecture rather than simple compromises. Natural components can provide ECM-like permissiveness or degradable motifs, synthetic networks can control mechanics and viscoelasticity, swelling and depot stability, and secondary modulus can deliver cytokines, antigens, antibodies, chemokines, adjuvants, or protease-responsive linkers [9,17,28,29,30]. How these components are incorporated is a central design variable. Physical entrapment is simple and cytocompatible but often causes burst release. Covalent conjugation improves retention but may reduce bioactivity or expose cells to reactive chemistry. Affinity-based interactions, including heparin-, cyclodextrin-, antibody-, or receptor-ligand-mediated retention, can prolong release while preserving reversible availability, whereas supramolecular interactions can support injectable recovery and modular cue exchange [31,32,33]. These incorporation strategies should match the intended biological timing, such as immediate activation, sustained survival support, delayed egress, or staged immune reprogramming. From a translational perspective, natural systems require rigorous source and lot qualification because of source variability and batch-to-batch heterogeneity, whereas synthetic systems generally offer greater compositional reproducibility and regulatory comparability owing to defined precursor chemistry. Hybrid systems can provide functional advantages but may increase quality-control and regulatory-comparability burdens because each component, interface, and intercomponent interaction must be controlled [22,24,28].

### 2.2. Cytocompatible Gelation and Crosslinking Strategies

The gelation route determines how cells experience encapsulation. It controls the loading window, precursor viscosity and osmolality, exposure to radicals or reactive intermediates, network homogeneity, and the rate of depot formation after administration [34,35]. For immune cell products, gelation should be evaluated according to cytocompatibility, reproducibility, and preservation of function rather than chemistry class alone [22,34,35,36].

Physically assembled, ionically crosslinked, and supramolecular hydrogels are often favored when mild processing and syringe delivery are the dominant priorities [37]. Because networks form through temperature change, ionic complexation, host-guest binding, hydrogen bonding, or other reversible interactions, cells can frequently be mixed under aqueous conditions with limited exposure to reactive chemistry [31]. The trade-off is that reversible networks may be more sensitive to dilution, ionic composition, or mechanical disruption and may therefore show less predictable long-term retention [13,32].

Covalently crosslinked hydrogels provide stable geometry and tunable mechanics, swelling, degradation, and ligand density [38,39]. They are useful when prolonged local retention or sustained co-delivery is required, but they also introduce chemistry-specific risks. Acrylate- or maleimide-based Michael-type reactions can interact with nucleophilic cell-surface thiols; aldehyde- or Schiff-base systems may alter cell-surface proteins if residual reactive groups persist; radical-mediated polymerization can damage lymphocytes through photoinitiator toxicity, radical diffusion, or excessive light exposure; and pH shifts, catalysts, unreacted precursors, or degradation products may alter immune cell phenotypes even when viability appears acceptable [36,38,39,40,41,42]. These concerns provide a practical rationale for using thiol-free or cell-orthogonal alternatives, such as enzymatic crosslinking, strain-promoted click chemistry, tetrazine-norbornene ligation, or affinity/supramolecular assembly, when encapsulating activated T-cells, NK cells, or other functional immune effectors [31,43,44]. Functional qualification should assess cytotoxicity, cytokine secretion, migration, immune-synapse formation, antigen presentation, and polarization rather than viability alone.

Enzymatic crosslinking occupies a useful middle ground. Horseradish peroxidase, transglutaminase, tyrosinase, and related biocatalytic systems can form networks under near-physiological aqueous conditions, but their reaction kinetics, enzyme source, lot-to-lot consistency, cost, and residual oxidant burden must be controlled [45,46]. Photo-crosslinking offers strong spatial and temporal control, including rapid curing and patterning, but immune cell applications require more extensive qualification than acellular biomaterials. Type I photoinitiators undergo direct photolysis to generate radicals, whereas type II systems require co-initiators or hydrogen abstraction; these mechanisms differ in radical yield, oxygen sensitivity, and cytotoxicity. Common biological hydrogel photoinitiators, such as Irgacure 2959 and lithium phenyl-2,4,6-trimethylbenzoylphosphinate (LAP) also differ in absorption range. Ultraviolet (UV) versus near-visible activation can therefore affect both cell viability and tissue penetration. Photocurable ACT formulations should report initiator identity and concentration, wavelength, light intensity, exposure time, curing depth, post-gelation residuals, and recovery of immune cell function [22,41].

### 2.3. Injectable Depots: In Situ-Forming, Shear-Thinning, and Self-Healing Hydrogels

Most ACT-relevant hydrogel chemistries can be adapted to injectable or in situ-forming depots. These formats are well suited to post-surgical cavities, resection margins, peritumoral injections, and other locoregional sites where rapid washout would reduce cell retention [31,43]. Before gelation, the precursor should remain injectable and maintain a homogeneous cell suspension. After administration, gelation should be rapid enough to prevent dispersion but not so rapid that premature gelation or prolonged precursor exposure compromises cell handling.

Shear-thinning and self-healing hydrogels address a key practical challenge: the network flows under shear and then rapidly recovers after exiting the needle [47]. However, injectability does not necessarily imply cytoprotection or clinical usability. Needle gauge, barrel diameter, syringe material, injection speed, extensional flow, interfacial stress, polymer concentration, cell density, and recovery kinetics can alter both injection force and cell stress [34,48]. Increasing polymer concentration often improves mechanical recovery and local retention but also increases precursor viscosity, which can impair cell mixing, loading homogeneity, and post-injection function. Injectable ACT depots should therefore report needle gauge, flow rate, injection force or pressure, route-specific injectability, post-extrusion cell distribution, and functional outcomes such as motility, cytotoxicity, cytokine secretion, immune-synapse formation, or antigen-presenting capacity rather than viability alone.

Taken together, hydrogel selection for living immune cell delivery should be framed as matching material design to cell-product requirements. The most useful formulation is not necessarily the most complex or biomimetic; it is the one that preserves cell competence while balancing injection performance, local retention, remodeling, cue presentation, and controlled cell release. Table 1 compares hydrogel platform classes and gelation strategies relevant to ACT. The cell-type-specific implications of these trade-offs are discussed in Section 4.

### 2.4. Fabrication and Preparation Workflow for Hydrogel-Cell Products

Fabrication of ACT hydrogel products should be described as a cell-processing workflow rather than solely in terms of polymer chemistry. Key steps include precursor synthesis and purification, sterilization or aseptic preparation, buffer exchange and osmolality adjustment, immune cell harvest and concentration, cell-polymer mixing, gelation or microgel assembly, syringe loading, administration, and post-delivery recovery. For bulk in situ-forming gels, the principal risks are premature gelation, nonuniform cell distribution, and prolonged exposure to reactive precursors. For preformed macroporous scaffolds, key concerns include surgical handling, cell-seeding efficiency, and local retention. For granular or microgel-based hydrogels, fabrication parameters such as microgel size distribution, particle stiffness, interstitial void fraction, annealing chemistry, and jamming density determine both injectability and the cell-scale spaces available for immune cell scanning [44,56,57,58,59,60].

Preparation methods should also be matched to the immune cell product. CAR-T and TCR-T products can be sensitive to overstimulation and prolonged handling; NK cells often require cytokine support during processing; DC products require preservation of antigen-presentation capacity and maturation status; and macrophage- or monocyte-derived products can change polarization in response to adhesion, stiffness, serum proteins, or inflammatory contaminants. Hydrogel-cell products should therefore be evaluated after the complete preparation sequence, including storage, mixing, injection, and recovery. A formulation that appears cytocompatible during static encapsulation may still impair function during clinically relevant handling.

## 3. Hydrogel Properties That Govern Immune Cell Fate

After delivery, immune cells experience hydrogel as a dynamic microenvironment rather than a passive carrier. The principal design and reporting categories are injectability/in situ gelation (introduced in Section 2.3), mechanics/viscoelasticity, porosity/mass transport, degradability/remodeling, cargo release kinetics/stimuli-responsiveness, bioadhesion/ECM mimicry, immunogenicity/immune shielding, and swelling/depot stability. Together, these properties regulate survival, motility, activation, cytotoxicity, antigen presentation, polarization, persistence, and release. They should be interpreted as interacting material levers that shape immune cell function and underpin the cell-type-specific framework in Section 4, as summarized in Table 2.

### 3.1. Mechanics/Viscoelasticity

Mechanical design should not be reduced to static stiffness. In three-dimensional (3D) hydrogels, immune cells experience confinement, stress relaxation, and force transmission as they scan, migrate, and form cell–cell contacts [30,62,63]. For lymphocytes, an overly confining or slowly relaxing matrix can impede motility and reduce target access even when viability is preserved [64]. By contrast, a network that is too weak may fail to retain cells or organize local stimulatory interactions. The relevant question is whether the hydrogel’s dynamic mechanics support the contact-dependent functions of the delivered cell product.

Stress relaxation is therefore often more informative than elastic modulus alone. Rapidly relaxing or dynamically bonded networks permit transient deformation and recovery, supporting amoeboid lymphocyte movement without sacrificing depot integrity [65,102,103]. Dynamic bond exchange may also shorten the duration of physical confinement on migrating cells. However, direct mechanistic links between specific viscoelastic parameters and T-cell exhaustion programs remain to be established.

Myeloid effectors follow a different mechanical logic. DCs and macrophages can undergo stiffness-sensitive metabolic and functional reprogramming, including changes in maturation, cytokine output, and polarization state [53,69]. Thus, mechanics may primarily preserve motility in lymphoid effectors but actively instruct myeloid effectors. No single stiffness or relaxation profile should be considered as optimal across ACT products. NK cells represent an intermediate case: they require motility-permissive mechanics for immune-synapse formation and cytolytic function, but their activating-receptor signaling is also sensitive to substrate stiffness [66].

### 3.2. Porosity/Mass Transport

Porosity and mass transport determine whether a hydrogel functions as a permissive immune niche or a diffusion-limited barrier. Pore size, interconnectivity, permeability, tortuosity, and effective diffusivity govern access to oxygen, nutrients, cytokines, chemokines, and waste removal [56,57]. For ACT, the material must support soluble-cue exchange while allowing therapeutic cells to redistribute or exit the depot when required.

Bulk hydrogels often face a structural tension: polymer concentrations that support mechanical integrity can generate nanoscale mesh sizes that limit cell transit and transport. Macroporous gels improve migration and diffusion, but many preformed or foamed structures are difficult to deliver through narrow needles [58]. Granular hydrogels address this problem by assembling jammed or annealed microgels into an injectable architecture. The interstitial voids between particles provide cell-scale space and mass transport, while the particulate network can shear-thin and reassemble after injection [58,59]. This design principle is directly supported by ex vivo evidence that T-cells remain trapped in dense crosslinked gelatin networks but recover full motility in foamed microgel architectures with optimized pore geometry [61].

Granular platforms also provide a high surface area for local cue presentation. Microgel surfaces can be functionalized with antibodies, ligands, cytokines, or artificial antigen-presenting signals so that migrating cells encounter instructive cues within an architecture that also permits transport [54,55,60,61]. Porosity is therefore not only a physical parameter but also a means of organizing immune stimulation. Void space, particle mechanics, polymer concentration, and surface chemistry should be tuned together rather than treated as independent variables. Increasing polymer concentration or particle packing may improve post-injection recovery and residence time, but it can also increase injection force, reduce void connectivity, and expose cells to greater mechanical stress during loading and extrusion. Porosity/mass transport and injectability/in situ gelation should therefore be reported together rather than as separate optimization criteria.

### 3.3. Degradability/Remodeling and Cargo Release Kinetics/Stimuli-Responsiveness

Degradability/remodeling controls depot lifetime, the timing of cell release, and the extent to which cells can modify their local environment [23,89]. For T- and NK-cell depots, degradation should not be treated simply as a migration-enabling feature because lymphocytes can move through permissive 3D spaces by amoeboid mechanisms without extensive proteolysis [78,79,80,81]. Instead, degradation primarily tunes early retention, later tissue infiltration, and access to the surrounding tumor. A stable depot may retain cells but limit infiltration, whereas a rapidly degrading depot may release cells before adequate local activation or expansion.

For DCs, macrophages, and monocyte-derived effectors, degradation can be more instructive. Cell-responsive or protease-cleavable linkers can couple matrix remodeling to local inflammatory activity, while degradation products from ECM-derived materials may influence maturation, cytokine output, or polarization [54,89,90]. For these products, both degradation rate and degradation-product identity should be considered.

Stimuli-responsive hydrogels extend this logic by coupling matrix behavior or cargo release kinetics to proteases, pH, reactive oxygen species (ROS), temperature, or other local triggers [96,97,98]. Their value lies not in being generically ‘smart’ but in matching a defined response, such as matrix softening, cell release, cytokine release, antigen exposure, or checkpoint-modulator delivery to relevant biological trigger. It should also describe the response chemistry. Protease-responsive matrices often use matrix metalloproteinase (MMP)-cleavable peptide crosslinkers; acidic pH-responsive systems may use hydrazone, acetal, imine, or boronate-based motifs; ROS-responsive systems often incorporate thioketal, boronic ester, thioether, selenium-, or tellurium-containing linkages; and thermo-responsive systems rely on lower critical solution temperature behavior or sol–gel transitions [90,96,97,98]. Because tumor extracellular pH and ROS levels vary across tumor types and regions, responsive chemistries should be validated under biologically relevant pH, oxidative, enzymatic, and diffusion gradients rather than inferred from accelerated bulk-release assays.

### 3.4. Bioadhesion/ECM Mimicry

Bioadhesion/ECM mimicry shapes retention, scanning, migration, and responses to locally presented cues [104]. However, greater biomimicry is not automatically better for ACT. Many therapeutic immune cells require rapid movement, transient adhesion, and context-dependent arrest. Too little adhesion can cause washout, whereas too much can trap cells, create nonproductive adhesion, or interfere with engineered effector function.

The design goal should therefore be function-selective mimicry rather than broad ECM replication. ECM-derived matrices or ligand-functionalized networks may be useful when adhesion, remodeling, or innate sensing is desired. Conversely, defined stimulation matrices that present a minimal set of immune ligands may better support lymphocyte activation, synapse-like interactions, or localized expansion [50]. These adhesive trade-offs across T-cells, NK cells, γδ T-cells, and myeloid effectors are discussed in Section 4. TILs occupy a distinct position: intratumoral residence induces expression of CD103 (integrin αE) expression through transforming growth factor-β (TGF-β) signaling, promoting adhesion to E-cadherin on epithelial tumor cells [91]. This integrin reprogramming may influence how TILs respond to synthetic or decellularized matrices after reintroduction, particularly when E-cadherin-like ligands are absent. For γδ T-cells, subset-specific tissue tropism involves αE and α1 integrins in epithelial-resident Vδ1 cells and distinct homing receptors in circulating Vδ2 cells [68]. These patterns suggest that depot ligand identity and density may differentially influence local retention and subsequent infiltration toward epithelial or stromal targets. Because direct hydrogel evidence for γδ T-cells is limited, the corresponding entries in Table 2 are explicitly presented as design hypotheses.

### 3.5. Immunogenicity/Immune Shielding

Hydrogels also create an immunological interface with the host. Protein adsorption, complement activation, innate immune recruitment, foreign-body responses, fibrosis, and degradation products can alter transport, cytokine gradients, cell survival, and depot permeability [17,28,36,49]. For ACT, the goal is not complete immunological neutrality. Local inflammation may support immune activation in some settings, whereas fibrotic encapsulation or uncontrolled innate activation can isolate the depot and impair therapeutic-cell function.

Immune shielding is similarly double-edged. Encapsulation can protect cells from inflammatory cytokines, allogeneic immune recognition, or rapid immune-mediated clearance and may extend persistence in hostile environments [105,106]. However, excessive shielding can reduce crosstalk with host tissue, limit beneficial recruitment, and create transport barriers. For allogeneic NK cells, this issue is especially important: the lower risk of graft-versus-host disease (GvHD) does not eliminate host rejection or limited persistence. Hydrogel lifetime and cytokine-release duration should therefore be matched to the expected window of NK-cell survival and activity [82,83,84,85,86,87,88]. ACT hydrogel design should accordingly aim for a calibrated interface that protects the cell product without biologically isolating it. Evaluation should include host–cell recruitment, fibrosis, depot permeability, cell mobilization, and retained effector function rather than the intrinsic immunogenicity of the material alone [23,105].

### 3.6. Swelling/Depot Stability

Swelling behavior and depot stability should be treated as a functional ACT design category rather than a purely physical property. After implantation or injection, water uptake can alter the effective modulus, pore size, local cytokine concentration, and cell density within the depot. In confined tumor beds or postsurgical cavities, excessive swelling may compress surrounding tissue, alter interstitial flow, or change the distance between immune cells and target cells. Insufficient hydration, by contrast, may restrict mass transport and reduce cell viability. Studies should report the definition of swelling ratio, equilibrium water content, and time-dependent volume change should therefore be reported under physiological ionic strength and serum-containing conditions, together with post-swelling mechanics and release behavior [23,35,89].

Chemical and structural stability should also be evaluated under conditions relevant to the intended in vivo use, including 37 °C, physiological ionic strength, serum-protein adsorption, enzymatic activity, inflammatory oxidants, and mechanical loading. Physically assembled or supramolecular gels may be sensitive to dilution and ionic exchange; covalent gels may be more stable but can accumulate stress or undergo hydrolysis; ECM-derived gels may be remodeled by host enzymes; and granular gels may change porosity as particles swell, degrade, or anneal. For ACT, depot stability should not simply be maximized. Instead, it should match the intended therapeutic sequence of cell retention, activation, migration, and tissue infiltration.

## 4. Cell-Type-Tailored Hydrogel Design and Effectiveness

### 4.1. T Cells (CAR-T/TCR-T/TIL)

Among the immune effector populations considered for ACT, T-cells remain the most extensively studied and clinically advanced [1,2]. Accordingly, hydrogel-based strategies have been developed most comprehensively for CAR-T cells, TCR-T cells, and TILs. The central design challenge is to preserve potent cytotoxic function while avoiding premature exhaustion and maintaining sufficient persistence in the hostile TME. In solid tumors, transferred T-cells often encounter limited antigen accessibility, poor infiltration, metabolic stress, and inhibitory signaling, all of which constrain efficacy [5,106,107].

Hydrogels can address these limitations by serving as localized depots that retain transferred T-cells together with supportive biochemical cues. Rather than acting as passive carriers, these systems create a confined microenvironment that enhances cell–cell interactions, improves engagement with antigen-presenting cells, and prolongs productive activation [10,30,108]. Representative studies have shown that implantable or injectable hydrogel systems can localize CAR-T cells at tumor sites while co-delivering cytokines or adjuvants, thereby improving tumor control and persistence [33,46,99,100].

Hydrogel design for T-cells must balance activation and exhaustion [101]. Sufficient stimulation is required for proliferation and effector differentiation, but excessive or prolonged signaling can induce dysfunction. Hydrogel architecture can therefore regulate T-cell fate [15]. An overly rigid or slowly degradable matrix may restrict migration and create a static niche, whereas a rapidly degrading matrix may fail to sustain local signaling. Rather than prescribing a universal optimum, T-cell-oriented hydrogels should provide context-appropriate mechanical support, permit remodeling, and enable dynamic interactions with surrounding tissue.

It is important to distinguish depot-style delivery from scaffold-based priming. Depot systems primarily retain cells and bioactive factors at the target site, whereas scaffolds can prime T-cells ex vivo or in situ by controlling activation, costimulation, and expansion. These strategies are not mutually exclusive and may be integrated to condition T-cells before supporting their persistence after delivery. Stiffness, porosity/mass transport, degradability/remodeling, and ligand presentation collectively influence migration, activation thresholds, and metabolic fitness [93]. T-cell-tailored hydrogels should therefore be viewed as tunable immunological niches that maintain effector function while limiting overstimulation-induced dysfunction.

### 4.2. NK Cells (CAR-NK)

NK cells are attractive alternatives to T-cells because they mediate rapid, antigen-independent cytotoxicity and carry a lower risk of GvHD [82,109]. However, NK-cell–based therapies have distinct limitations, including limited in vivo persistence, modest expansion, and rapid functional decline after infusion [83]. Many clinically developed CAR-NK products are allogeneic and remain susceptible to host-mediated rejection despite their lower GvHD risk. These features create engineering requirements that differ from those of CAR-T therapies.

Hydrogels can provide a supportive 3D niche that enhances NK-cell viability and preserves cytotoxic function [84]. A key requirement is local cytokine support, particularly interleukin-15 (IL-15), which promotes NK-cell survival, proliferation, and activation [85,86]. Because systemic IL-15 can cause toxicity, localized and controlled delivery is desirable [87]. Hydrogel systems can combine an initial activation phase with sustained cytokine presentation to support function over time.

Compared with CAR-T cells, whose persistence often depends on maintaining memory-like phenotypes, CAR-NK cells rely more heavily on continued cytokine support. Spatiotemporally controlled cytokine presentation is therefore a central design feature for NK-cell-directed hydrogels [110,111]. The cell source also matters, since many CAR-NK products are used as off-the-shelf allogeneic therapies [112,113]; hydrogel depots may consequently need to balance local retention and cytokine duration against the anticipated in vivo persistence of the selected NK-cell product. These differences underscore why CAR-T hydrogel design principles cannot be directly extrapolated to CAR-NK therapy.

Biophysical design is equally important. NK-cell cytotoxicity depends on immune-synapse formation, cell mobility, and repeated target contact [88]. Because NK cells form short-lived, dynamic synapses during serial killing, matrices that permit rapid migration and repeated interactions may be especially important [84,110,114]. An overly restrictive matrix may impede migration, whereas an overly permissive matrix may fail to retain cells at the tumor site. NK-cell-tailored hydrogels should therefore combine local retention with dynamic permissiveness.

Hydrogel-based systems can enhance NK-cell antitumor activity by integrating cytokine delivery with mechanical and biochemical cues that preserve the cytotoxic phenotype [17]. Biomaterial niches have also been reported to improve NK-cell survival and functional persistence [84]. CAR-NK hydrogels should therefore be designed as supportive microenvironments that compensate for limited persistence while preserving serial killing in immunosuppressive tumors. Compared with CAR-T-oriented systems, their priorities may place greater emphasis on cytokine support, local retention, and repeated target engagement.

### 4.3. γδ T-Cells

Conventional αβ T-cell therapies include major histocompatibility complex (MHC)-restricted TCR-T cells and MHC-independent CAR-T cells. γδ T-cells have attracted translational interest because their innate-like recognition can also operate largely independently of classical MHC presentation and because allogeneic products are associated with a low risk of GvHD [115,116]. Their use against solid tumors is nevertheless limited by suboptimal tissue homing, functional decline within hostile tumor cores, and subset-specific microenvironmental vulnerabilities [117].

Hydrogel-enabled delivery for γδ T-cells should therefore be framed as a hypothesis-driven design problem rather than an established engineering field. Distinct γδ T-cell subsets differ in integrin expression and trafficking, which may influence the balance between local retention and tissue infiltration [118]. Epithelial-resident Vδ1+ cells can express αEβ7 and α1β1 integrins that support interactions with E-cadherin and collagen-rich tissues [119,120]. Depots intended for Vδ1+ products may therefore benefit from defined, low-density adhesive motifs or selected ECM-derived components that limit washout without restricting amoeboid migration.

Circulating Vγ9Vδ2+ cells can be recruited through chemokine axes that include C-X-C chemokine receptor 3 (CXCR3) and C-C chemokine receptor 5 (CCR5) [121]. For these products, hybrid hydrogels could be investigated for sustained presentation of matching chemokines, such as CXCL9 or CXCL10, or aminobisphosphonates such as zoledronic acid, which can promote phosphoantigen accumulation in target cells [121,122]. Because direct biomaterial studies specifically investigating hydrogel-mediated γδ T-cell delivery remain limited, no consensus hydrogel design principles have yet been established for this cell type. Therefore, the matrix-matching strategies discussed above are presented as hypothesis-generating concepts derived from current knowledge of γδ T-cell biology and tissue trafficking, rather than evidence-based engineering rules.

### 4.4. Dendritic Cells and Macrophage/Monocyte-Derived Effectors

DCs and macrophage/monocyte-derived effectors are functionally distinct but complementary components of the immune system and require different hydrogel design logic from cytotoxic lymphocytes [67,73,123]. Whereas T- and NK-cells act primarily through direct tumor-cell killing, these myeloid cells regulate immune activation and tissue-level organization. Hydrogels for these populations should therefore do more than support survival; they should modulate immune signaling, differentiation, and microenvironmental dynamics.

For DCs, the primary objective is to enhance antigen presentation and subsequent T-cell priming. Hydrogels can co-localize antigens and immunostimulatory signals, increasing the likelihood that DCs encounter antigen under activating rather than tolerogenic conditions [92]. Biomaterial-based vaccine platforms have shown that co-delivery of antigen and adjuvant can enhance DC maturation and downstream T-cell responses [74,75,76].

Cryogel- and alginate-based vaccine depots have also promoted DC recruitment, maturation, and lymph-node trafficking through localized delivery of granulocyte-macrophage colony-stimulating factor (GM-CSF), CpG oligonucleotides, and stimulator of interferon genes (STING) agonists [124,125]. Temporally controlled release can further coordinate early antigen uptake with sustained exposure to maturation signals [126,127]. Hydrogels may thus function as transient educational niches that organize antigen loading, activation, and controlled DC egress.

Macrophage- and monocyte-derived effectors are more directly involved in remodeling the TME. In many tumors, macrophages adopt immunosuppressive phenotypes that promote progression, angiogenesis, and immune evasion [77,94]. Hydrogel-based strategies therefore aim to reprogram these cells toward pro-inflammatory, antitumor states [70,95]. Cytokines, pattern-recognition ligands, and other immunomodulators can be incorporated into the matrix for local presentation [128]. Preclinical hydrogel systems containing interferon-γ (IFN-γ), Toll-like receptor (TLR) agonists, or STING agonists have been reported to reprogram tumor-associated macrophages and improve cytotoxic lymphocyte infiltration [125].

Macrophage behavior is highly sensitive to biochemical and biophysical cues. Matrix stiffness, crosslinking density, degradability/remodeling, and ECM-mimetic components can alter macrophage activation and cytokine production [71,95]. Hydrogels are therefore active regulators of innate immune function rather than passive delivery vehicles in this context. By favoring inflammatory activation, they may help reshape the TME, recruit immune cells, and improve combination therapies.

Monocyte-derived effectors, particularly monocyte-derived dendritic cells (moDCs) and monocyte-derived macrophages (moMDMs), are highly plastic and transition through progressive functional and structural states after tissue infiltration [129,130]. Their delivery requires a design paradigm distinct from that used for cytotoxic lymphocytes. Monocytes and early differentiated progeny sense substrate stiffness and spatial confinement, which can alter metabolism, receptor expression, and cytokine secretion [131]. For products intended to remodel an immunosuppressive TME, the hydrogel should balance mechanical feedback with biochemical signaling. Dense or highly confining networks may activate YAP/TAZ- and PIEZO1-associated mechanosensitive pathways, which can influence differentiation and potentially favor suppressive phenotypes [132,133].

Monocyte-based ACT platforms may therefore benefit from granular or interconnected macroporous hydrogels that provide cell-scale voids for spreading and elongation, morphologies associated with pro-inflammatory activation and altered cytokine production [134,135]. These matrices can also serve as controlled-release depots for TLR agonists, GM-CSF, or IFN-γ [124,125]. Coordinating biophysical and biochemical inputs may promote in situ differentiation of transferred monocytes toward inflammatory and antigen-presenting phenotypes while reducing susceptibility to tumor-derived suppressive signals.

Collectively, hydrogels for DCs and macrophage/monocyte-derived effectors should be regarded as immune-orchestrating platforms that regulate antigen presentation, innate immune polarization, and local recruitment. Unlike hydrogels for cytotoxic lymphocytes, these systems act primarily by remodeling the immune microenvironment and coordinating multicellular interactions that support durable antitumor responses.

## 5. Limitations and Future Perspectives

Despite the therapeutic potential of cell-type-tailored hydrogels, technical, regulatory, and translational challenges must be addressed before broad clinical implementation. Sterility assurance and bioprocess compatibility are particularly important. Terminal sterilization methods, including autoclaving, ethylene oxide treatment, and high-dose γ-irradiation, are generally incompatible with living cell-laden hydrogel products because they can alter mechanics, degradability/remodeling, crosslinking networks, and ligand presentation while compromising cell viability and function [136,137]. Manufacturing therefore relies on aseptic processing, pre-sterilized components, sterile filtration of polymer precursors when feasible, and closed-system workflows. These requirements increase the complexity of process standardization, automation, and scale-up.

Shelf life, cryopreservation, and cold-chain logistics are additional barriers. Unlike acellular biomaterials, cell-laden hydrogels contain metabolically active immune cells whose viability and function may decline during storage and transport [138,139]. Integration into centralized manufacturing will require preservation strategies that maintain both cellular integrity and hydrogel stability.

Another limitation is the lack of quantitative design rules for rational hydrogel engineering. Many studies describe materials qualitatively as soft, stiff, or rapidly degradable, which limits cross-study comparison [140]. Future research should systematically establish quantitative relationships between hydrogel properties, including elastic modulus, pore size, degradation kinetics, swelling behavior, and cytokine release profiles, and specific immune cell functions such as persistence, migration, activation, and cytotoxicity. Standardized reporting of these parameters will be essential for improving reproducibility and facilitating the development of predictive design principles.

Future hydrogel platforms should also incorporate advanced functionalities that enable real-time monitoring and adaptive therapeutic responses. For example, the integration of non-invasive imaging modalities, including magnetic resonance imaging (MRI), positron emission tomography (PET), and near-infrared fluorescence probes, may allow longitudinal assessment of depot degradation, immune cell persistence, and biodistribution [141]. Such approaches could provide valuable insights into the spatiotemporal behavior of hydrogel-assisted ACT in vivo and accelerate translational development.

Combination ACT represents another opportunity. Most current hydrogel systems deliver a single immune cell population, yet durable antitumor responses often depend on coordinated interactions among multiple cell types. Hydrogels can organize immune cells and immunomodulators within a localized niche with defined spatial and temporal regulation [128]. Future platforms could support sequential or simultaneous delivery of components such as DC vaccines with CAR-T cells, localized cytokine depots for CAR-NK cells, or macrophage-mediated TME reprogramming before cytotoxic-cell administration. Multiphase or compartmentalized hydrogels capable of orchestrating these interactions are an important direction for next-generation ACT.

Finally, several emerging immune cell platforms warrant further investigation. In particular, γδ T-cells have attracted increasing interest because of their MHC-independent recognition mechanisms and low risk of GvHD [142]. However, direct evidence regarding hydrogel-assisted γδ T-cell delivery remains extremely limited, and quantitative design principles specific to these cells have yet to be established [143]. Future studies should determine how mechanics/viscoelasticity, bioadhesion/ECM mimicry, chemokine presentation, and injectability/in situ gelation influence γδ T-cell persistence and antitumor activity.

Collectively, future hydrogel platforms for ACT will likely evolve from simple cell-delivery depots into dynamically engineered immunological niches capable of regulating cell survival, activation, migration, and intercellular communication in a spatiotemporally controlled manner. Achieving this goal will require the integration of immunology, biomaterials science, manufacturing engineering, and translational medicine to establish robust and clinically actionable design principles for hydrogel-enabled cell therapies.

## 6. Conclusions and Outlook

Hydrogel-enabled ACT is emerging as a practical strategy for addressing insufficient local retention, limited persistence, and functional suppression in solid-tumor immunotherapy. Hydrogels should be treated as immune-instructive microenvironments rather than generic carriers. For living immune cell products, the hydrogel platform and gelation route must preserve cell competence during loading and injection, support transport after implantation, and balance early retention with timely redistribution. These requirements highlight the translational value of cytocompatible, injectable formats, including in situ-forming depots and shear-thinning/self-healing matrices.

Immune cell function is governed by interacting hydrogel categories, including injectability/in situ gelation, mechanics/viscoelasticity, porosity/mass transport, degradability/remodeling, cargo release kinetics/stimuli-responsiveness, bioadhesion/ECM mimicry, immunogenicity/immune shielding, and swelling/depot stability. Their effects are cell-type-dependent. Lymphoid products, including CAR-T, TCR-T, TIL, NK, and CAR-NK cells, require motility-permissive niches that sustain activation without confinement-driven dysfunction. Myeloid products, including DCs and macrophage/monocyte-derived effectors, require matrices that coordinate recruitment, maturation, polarization, and immune organization. These differences support a cell-type-tailored design framework.

Future progress will depend on standardized reporting, function-first endpoints beyond viability, head-to-head comparisons under matched conditions, and early integration of manufacturing, sterilization, handling, and safety considerations. Aligning hydrogel design with the biology of specific immune cell products should accelerate the development and translation of safer and more effective biomaterial-assisted ACT platforms.

## Figures and Tables

**Table 1:** Comparative summary of hydrogel platform classes and gelation strategies for ACT.

Category	Main Strength	Cytocompatibility	Manufacturing	References
Platform Class				
**Natural** (collagen, fibrin, etc.)	ECM-like cues; intrinsic remodeling signals relevant to DCs and macrophages	Often supports high initial viability; performance may vary for CAR-T cells because of nonproductive adhesion	Source and batch variability; impurity, endotoxin, source control, sterilization, and lot-qualification controls are critical	[22,23,24,25,49]
**Synthetic** (PEG, polyacrylamide, etc.)	Compositional control; modular functionalization	Biologically inert unless functionalized; useful for controlled T- or NK-cell stimulation and hypothesis-driven mechanobiology	High reproducibility and GMP compatibility; defined precursor purity	[9,22,26,27,32,50]
**Hybrid** (natural + synthetic)	Decouples cytocompatibility from mechanics and cue delivery	Suitable for multiple cell products; balances permissiveness and control	Greater component complexity; each component and intercomponent interaction require GMP qualification	[9,17,28,29,30,33,43,51,52,53]
**Gelation Strategy**				
**Physical/** **I** **onic** **/supramolecular**	Mild aqueous loading; syringe-injectable	Low reactive burden; long-term stability and retention may vary	Excellent injectability; formulation stability may vary	[31,32,33,37,47]
**Chemical** **/** **C** **ovalent**	Stable geometry; tunable mechanics, swelling, and degradation	Residual reactants and pH shift require screening; thiol-reactive precursors may alter T- or NK-cell surface chemistry	Good process control once the chemistry is qualified	[38,39,40,41,42,43,44]
**Enzymatic**	Near-physiological gelation; avoids UV light and photo initiators	Cell-orthogonal options can minimize surface-thiol interferences; useful for activated T and NK cells	Enzyme source, lot, cost, and oxidant burden must be controlled	[45,46]
**Photo-crosslinking**	Rapid curing; spatial and temporal precision	Photo initiator toxicity and radical diffusion require rigorous qualification in lymphocytes	Requires optical workflow and thorough functional testing needed	[22,39]
**Granular** **/Microporous** **fabrication**	Cell-scale voids, high surface area for cue presentation, shear-thinning injection, and migration-permissive architecture	Particularly useful for motile lymphocytes and for DC/macrophage recruitment or release	Microgel size, stiffness, void fraction, jamming density, and annealing chemistry should be reported	[44,54,55,56,57,58,59,60]

**Table 2:** Hydrogel property categories and cell-type-specific functional consequences for ACT.

Property	T Cells (CAR-T/TCR-T/TIL)	NK Cells/CAR-NK	γδ T Cells	Monocyte-Derived Effectors
**Injectability** **/In** **situ gelation**	Preserve viability, motility, and cytotoxicity; avoid overstimulation or reactive exposure [34,41,43,48]	Minimize shear stress while maintaining retention and mobility [34,48,51,52]	Likely follows lymphocyte requirements; direct injection data are limited	Preserve antigen presentation or polarization during gelation and injection [22,34,35,36]
**Mechanics** **/V** **iscoelasticity**	Rapid stress relaxation and low confinement support scanning and target contact [61,62,63,64,65]	Motility-permissive mechanics support synapse formation and serial killing [66,67]	Likely requires lymphocyte-like deformability; direct hydrogel evidence is scarce [68].	Mechanics can regulate DC maturation, cytokine output, and macrophage polarization [53,69,70,71,72]
**Porosity** **/M** **ass** **transport**	Macroporous or granular structures support migration, expansion, and tumor-cell contact [54,55,56,57,58,59,61]	Interconnected voids support scanning, serial killing, and cytokine access [51,52]	Cell-scale connected paths may support tissue surveillance; thresholds are unknown	Pore architecture regulates antigen/adjuvant access, recruitment, maturation, and release [56,73,74,75,76,77]
**Degradability** **/** **remodeling**	Early retention with controlled release is preferred; proteolysis is not always required [78,79,80,81].	Stable local support with later redistribution may compensate for short persistence [82,83,84,85,86,87,88]	Subset-specific residence and infiltration requirements remain hypothetical [68]	Degradation products and responsive linkers can modulate innate sensing and polarization [44,54,70,71,89,90]
**Bioadhesion** **/** **ECM mimicry**	Defined or minimal adhesion can prevent nonproductive trapping while supporting activation [32,50,55]	Transient adhesion may support synapse formation; excessive ECM mimicry may limit scanning [66,67]	Ligand identity may affect Vδ1/Vδ2 retention and infiltration [68,91]	ECM-derived or defined ligands can guide maturation, recruitment, and phenotype control [70,71,72,73,74,75,76,77,92]
**Immunogenicity** **/** **Immune** **shielding**	Fibrosis can limit cell release and permeability; stimulation must avoid exhaustion [36,49,93]	Inflammation may activate NK cells but can also accelerate allogeneic rejection [82,83,84,85,86,87,88]	General lymphocyte logic applies; hydrogel-specific host-interface data are limited	Local inflammatory tone shapes DC maturation and macrophage/monocyte polarization [20,36,49,70,71,72,94,95]
**Cargo release kinetics** **/** **Stimuli** **-** **responsiveness**	Responsive release can coordinate activation, cytokine support, and checkpoint modulation [43,96,97,98,99,100,101]	Staged cytokine delivery may provide early activation and sustained IL-15/IL-2 support [52,86,87,88]	Responsive local release may be useful, but evidence remains limited	Antigen, adjuvant, and reprogramming cues should match recruitment and maturation windows [70,71,72,73,74,75,76,77,92]
**Swelling** **/** **Depot** **stability**	Excess swelling can dilute cell–cell contact and delay release [23,35,89]	Swelling can alter cytokine concentration, retention, and persistence	No direct hydrogel design rules are currently available	Swelling and degradation can alter tissue pressure, antigen diffusion, and innate-cell recruitment [23,49,60]

## Data Availability

No new data were created or analyzed in this study.

## References

[1] June C.H., O’Connor R.S., Kawalekar O.U., Ghassemi S., Milone M.C. (2018). CAR T cell immunotherapy for human cancer. Science.

[2] Patel K.K., Tariveranmoshabad M., Kadu S., Shobaki N., June C. (2025). From concept to cure: The evolution of CAR-T cell therapy. Mol. Ther..

[3] Fesnak A.D., June C.H., Levine B.L. (2016). Engineered T cells: The promise and challenges of cancer immunotherapy. Nat. Rev. Cancer.

[4] Newick K., O’Brien S., Moon E., Albelda S.M. (2017). CAR T Cell Therapy for Solid Tumors. Annu. Rev. Med..

[5] Rafiq S., Hackett C.S., Brentjens R.J. (2020). Engineering strategies to overcome the current roadblocks in CAR T cell therapy. Nat. Rev. Clin. Oncol..

[6] Hou A.J., Chen L.C., Chen Y.Y. (2021). Navigating CAR-T cells through the solid-tumour microenvironment. Nat. Rev. Drug Discov..

[7] Bonifant C.L., Jackson H.J., Brentjens R.J., Curran K.J. (2016). Toxicity and management in CAR T-cell therapy. Mol. Ther. Oncolytics.

[8] Mohammadzadeh V., Atapour-Mashhad H., Shahvali S., Salehi B., Shaban M., Shirzad M., Salahvarzi A., Mohammadi M. (2025). Hydrogels as advanced drug delivery platforms for cancer immunotherapy: Promising innovations and future outlook. J. Nanobiotechnol..

[9] Chen X., Wu S., Zhang Y., Feng L., Chen Y., Yang X., Men K., Zhu J., Li M. (2026). Hydrogels for Cancer Immunotherapy: Strategies From Construction to Application. MedComm.

[10] Caliari S.R., Burdick J.A. (2016). A practical guide to hydrogels for cell culture. Nat. Methods.

[11] Liu Y., Liu F., Zeng Y., Lin L., Yu H., Zhang S., Yang W. (2024). Hydrogel systems for spatiotemporal controlled delivery of immunomodulators: Engineering the tumor immune microenvironment for enhanced cancer immunotherapy. Front. Cell Dev. Biol..

[12] Tian S., Yang S., Liu Y. (2026). Hydrogel-based drug delivery systems for enhanced tumor therapy. RSC Adv..

[13] Liu C., Liao Y., Liu L., Xie L., Liu J., Zhang Y., Li Y. (2023). Application of injectable hydrogels in cancer immunotherapy. Front. Bioeng. Biotechnol..

[14] Mohaghegh N., Ahari A., Zehtabi F., Buttles C., Davani S., Hoang H., Tseng K., Zamanian B., Khosravi S., Daniali A. (2023). Injectable hydrogels for personalized cancer immunotherapies. Acta Biomater..

[15] Discher D.E., Janmey P., Wang Y.L. (2005). Tissue cells feel and respond to the stiffness of their substrate. Science.

[16] Chaudhuri O., Gu L., Klumpers D., Darnell M., Bencherif S.A., Weaver J.C., Huebsch N., Lee H.P., Lippens E., Duda G.N. (2016). Hydrogels with tunable stress relaxation regulate stem cell fate and activity. Nat. Mater..

[17] Dravid A.A., Singh A., Garcia A.J. (2025). Biomaterial-Based Therapeutic Delivery of Immune Cells. Adv. Healthc. Mater..

[18] Rezvani K., Rouce R., Liu E., Shpall E. (2017). Engineering Natural Killer Cells for Cancer Immunotherapy. Mol. Ther..

[19] Palucka K., Banchereau J. (2012). Cancer immunotherapy via dendritic cells. Nat. Rev. Cancer.

[20] Mantovani A., Marchesi F., Malesci A., Laghi L., Allavena P. (2017). Tumour-associated macrophages as treatment targets in oncology. Nat. Rev. Clin. Oncol..

[21] Irvine D.J., Dane E.L. (2020). Enhancing cancer immunotherapy with nanomedicine. Nat. Rev. Immunol..

[22] Farasati Far B., Safaei M., Nahavandi R., Gholami A., Naimi-Jamal M.R., Tamang S., Ahn J.E., Ramezani Farani M., Huh Y.S. (2024). Hydrogel Encapsulation Techniques and Its Clinical Applications in Drug Delivery and Regenerative Medicine: A Systematic Review. ACS Omega.

[23] Lu P., Ruan D., Huang M., Tian M., Zhu K., Gan Z., Xiao Z. (2024). Harnessing the potential of hydrogels for advanced therapeutic applications: Current achievements and future directions. Signal Transduct. Target. Ther..

[24] Segneanu A.E., Bejenaru L.E., Bejenaru C., Blendea A., Mogosanu G.D., Bita A., Boia E.R. (2025). Advancements in Hydrogels: A Comprehensive Review of Natural and Synthetic Innovations for Biomedical Applications. Polymers.

[25] Das S.S., Sharma D., Rao B.V.K., Arora M.K., Ruokolainen J., Dhanka M., Singh H., Kesari K.K. (2023). Natural cationic polymer-derived injectable hydrogels for targeted chemotherapy. Mater. Adv..

[26] Kirkpatrick B.E., Dhand A.P., Hibbard L.P., Jaeschke M.W., Yendamuri T., Nelson B.R., Lee J.S., Bera K., Zlotnick H.M., Fox C.A. (2026). Ultrafast-relaxing and photopolymerizable PEG hydrogels enable viscoelasticity-mediated cell remodeling in synthetic matrices. Matter.

[27] van Holthe N.J.W., Wang Z., Lauko J., Gilbert E.P., Gangasandra V., Rowan A.E. (2024). Controlling ligand density and viscoelasticity in synthetic biomimetic polyisocyanide hydrogels for studying cell behaviours: The key to truly biomimetic hydrogels. Mater. Adv..

[28] Bu W., Wu Y., Ghaemmaghami A.M., Sun H., Mata A. (2022). Rational design of hydrogels for immunomodulation. Regen. Biomater..

[29] Xue Y., Che J., Ji X., Li Y., Xie J., Chen X. (2022). Recent advances in biomaterial-boosted adoptive cell therapy. Chem. Soc. Rev..

[30] Adu-Berchie K., Brockman J.M., Liu Y., To T.W., Zhang D.K.Y., Najibi A.J., Binenbaum Y., Stafford A., Dimitrakakis N., Sobral M.C. (2023). Adoptive T cell transfer and host antigen-presenting cell recruitment with cryogel scaffolds promotes long-term protection against solid tumors. Nat. Commun..

[31] Rodell C.B., MacArthur J.W., Dorsey S.M., Wade R.J., Wang L.L., Woo Y.J., Burdick J.A. (2015). Shear-Thinning Supramolecular Hydrogels with Secondary Autonomous Covalent Crosslinking to Modulate Viscoelastic Properties In Vivo. Adv. Funct. Mater..

[32] Weiden J., Voerman D., Dölen Y., Das R., Duffelen A., Hammink R., Eggermont L., Rowan A., Tel J., Figdor C. (2018). Injectable biomimetic hydrogels as tools for efficient T cell expansion and delivery. Front. Immunol..

[33] Appel E.A., Tibbitt M.W., Webber M.J., Mattix B.A., Veiseh O., Langer R. (2015). Self-assembled hydrogels utilizing polymer-nanoparticle interactions. Nat. Commun..

[34] Young S.A., Riahinezhad H., Amsden B.G. (2019). In situ-forming, mechanically resilient hydrogels for cell delivery. J. Mater. Chem. B.

[35] Li J., Mooney D.J. (2016). Designing hydrogels for controlled drug delivery. Nat. Rev. Mater..

[36] Fernandez-Yague M.A., Hymel L.A., Olingy C.E., McClain C., Ogle M.E., Garcia J.R., Minshew D., Vyshnya S., Lim H.S., Qiu P. (2022). Analyzing immune response to engineered hydrogels by hierarchical clustering of inflammatory cell subsets. Sci. Adv..

[37] Zhou H., Zheng H., Yao W., Sun H., Yang Y.G., Li Z., Song D., Zhang Y., Sun T. (2025). Spatiotemporally controlled delivery of biomacromolecules via injectable hydrogels for precision modulation of the tumor immune microenvironment. J. Nanobiotechnol..

[38] Sojdeh S., Panjipour A., Yaghmour A., Arabpour Z., Djalilian A.R. (2025). Click Chemistry-Based Hydrogels for Tissue Engineering. Gels.

[39] Carrascal-Hernandez D.C., Grande-Tovar C.D., Insuasty D., Marquez E., Mendez-Lopez M. (2026). Versatility of Click Chemistry in Hydrogel Synthesis: From Molecular Strategies to Applications in Regenerative Medicine. Gels.

[40] Lawrence D.A., Song R., Webert P. (1996). Surface thiols of human lymphocytes and their changes after in vitro and in vivo activation. J. Leukoc. Biol..

[41] Zhou W., Lei S., Liu M., Li D., Huang Y., Hu X., Yang J., Li J., Fu M., Zhang M. (2022). Injectable and photocurable CAR-T cell formulation enhances the anti-tumor activity to melanoma in mice. Biomaterials.

[42] Pellom S.T., Michalek R.D., Crump K.E., Langston P.K., Juneau D.G., Grayson J.M. (2013). Increased cell surface free thiols identify effector CD8+ T cells undergoing T cell receptor stimulation. PLoS ONE.

[43] Grosskopf A.K., Labanieh L., Klysz D.D., Roth G.A., Xu P., Adebowale O., Gale E.C., Jons C.K., Klich J.H., Yan J. (2022). Delivery of CAR-T cells in a transient injectable stimulatory hydrogel niche improves treatment of solid tumors. Sci. Adv..

[44] Song R., Murphy M., Li C., Ting K., Soo C., Zheng Z. (2018). Current development of biodegradable polymeric materials for biomedical applications. Drug Des. Devel Ther..

[45] Khanmohammadi M., Dastjerdi M.B., Ai A., Ahmadi A., Godarzi A., Rahimi A., Ai J. (2018). Horseradish peroxidase-catalyzed hydrogelation for biomedical applications. Biomater. Sci..

[46] Naranjo-Alcazar R., Bendix S., Groth T., Ferrer G.G. (2023). Research Progress in Enzymatically Cross-Linked Hydrogels as Injectable Systems for Bioprinting and Tissue Engineering. Gels.

[47] Esther J.M., Solanki R., Dhanka M., Thareja P., Bhatia D. (2024). Self-healing, injectable chitosan-based hydrogels: Structure, properties and biological applications. Mater. Adv..

[48] Calafel M.I., Criado-Gonzalez M., Aguirresarobe R., Fernández M., Mijangos C. (2025). From rheological concepts to additive manufacturing assessment of hydrogel-based materials for advanced bioprinting applications. Mater. Adv..

[49] Salthouse D., Novakovic K., Hilkens C.M., Ferreira A.M. (2023). Interplay between biomaterials and the immune system: Challenges and opportunities in regenerative medicine. Acta Biomater..

[50] Anandasivam N., Ali R., Gustinvil L., Rosenwasser M.J., Dunlop I.E., Delcassian D. (2026). Tunable Enhancement of T Cell Expansion Through Modulation of Stiffness and Adhesion Receptor Engagement in an Engineered Hydrogel Platform. Adv. Mater..

[51] Kim D., Jo S., Lee D., Kim S.M., Seok J.M., Yeo S.J., Lee J.H., Lee J.J., Lee K., Kim T.D. (2023). NK cells encapsulated in micro/macropore-forming hydrogels via 3D bioprinting for tumor immunotherapy. Biomater. Res..

[52] Jiao D., Hao M., Sun R., Ren X., Wei Y., Ding M., Yue X., Wu Z., Li C., Gao L. (2024). Dynamic Hybrid Module-Driven NK Cell Stimulation and Release for Tumor Immunotherapy. Nano Lett..

[53] Zhang Y., Liu Z., Kong W., Ao Y., Zhang R., Du Y. (2026). Viscoelastic hydrogel primed CAR-macrophage for pulmonary fibrosis treatment. Nat. Commun..

[54] Bhatta R., Han J., Liu Y., Bo Y., Wang H. (2023). T cell-responsive macroporous hydrogels for in situ T cell expansion and enhanced antitumor efficacy. Biomaterials.

[55] Lou J.Z., Meyer C., Vitner E.B., Adu-Berchie K., Dacus M.T., Bovone G., Chen A.Q., To T., Weitz D.A., Mooney D.J. (2024). Surface-Functionalized Microgels as Artificial Antigen-Presenting Cells to Regulate Expansion of T Cells. Adv. Mater..

[56] Wu C., Zhang H., Guo Y., Sun X., Hu Z., Teng L., Zeng Z. (2024). Porous Hydrogels for Immunomodulatory Applications. Int. J. Mol. Sci..

[57] Solbu A.A., Caballero D., Damigos S., Kundu S.C., Reis R.L., Halaas O., Chahal A.S., Strand B.L. (2023). Assessing cell migration in hydrogels: An overview of relevant materials and methods. Mater. Today Bio.

[58] Muir V.G., Qazi T.H., Shan J., Groll J., Burdick J.A. (2021). Influence of Microgel Fabrication Technique on Granular Hydrogel Properties. ACS Biomater. Sci. Eng..

[59] Griffin D.R., Archang M.M., Kuan C.H., Weaver W.M., Weinstein J.S., Feng A.C., Ruccia A., Sideris E., Ragkousis V., Koh J. (2021). Activating an adaptive immune response from a hydrogel scaffold imparts regenerative wound healing. Nat. Mater..

[60] Widener A.E., Roberts A., Phelps E.A. (2024). Granular Hydrogels for Harnessing the Immune Response. Adv. Healthc. Mater..

[61] Song Y., An S., Choi Y., Shin R., Song K.H., Doh J. (2026). Jammed Foamed Microgel-based Bioprinting for Ex Vivo Reconstruction of 3D T Cell-Cancer Cell Interactions. Adv. Healthc. Mater..

[62] Majedi F.S., Hasani-Sadrabadi M.M., Thauland T.J., Li S., Bouchard L.S., Butte M.J. (2020). T-cell activation is modulated by the 3D mechanical microenvironment. Biomaterials.

[63] Joseph J.P., Gugulothu S.B., Nandi D., Chatterjee K. (2023). Mechanical Properties Affect Primary T Cell Activation in 3D Bioprinted Hydrogels. ACS Macro Lett..

[64] Sapudom J., Alatoom A., Tipay P.S., Teo J.C. (2025). Matrix stiffening from collagen fibril density and alignment modulates YAP-mediated T-cell immune suppression. Biomaterials.

[65] Chaudhuri O., Cooper-White J., Janmey P.A., Mooney D.J., Shenoy V.B. (2020). Effects of extracellular matrix viscoelasticity on cellular behaviour. Nature.

[66] Yanamandra A.K., Zhang J., Montalvo G., Zhou X., Biedenweg D., Zhao R., Sharma S., Hoth M., Lautenschlager F., Otto O. (2024). PIEZO1-mediated mechanosensing governs NK-cell killing efficiency and infiltration in three-dimensional matrices. Eur. J. Immunol..

[67] Orange J.S. (2008). Formation and function of the lytic NK-cell immunological synapse. Nat. Rev. Immunol..

[68] Lawand M., Déchanet-Merville J., Dieu-Nosjean M.C. (2017). Key Features of Gamma-Delta T-Cell Subsets in Human Diseases and Their Immunotherapeutic Implications. Front. Immunol..

[69] Chakraborty M., Chu K., Shrestha A., Revelo X.S., Zhang X., Gold M.J., Khan S., Lee M., Huang C., Akbari M. (2021). Mechanical Stiffness Controls Dendritic Cell Metabolism and Function. Cell Rep..

[70] Butenko S., Nagalla R.R., Guerrero-Juarez C.F., Palomba F., David L.-M., Nguyen R.Q., Gay D., Almet A.A., Digman M.A., Nie Q. (2024). Hydrogel crosslinking modulates macrophages, fibroblasts, and their communication, during wound healing. Nat. Commun..

[71] Li W., Ye Q., Jiang Z., Xia D., Yan Z., Wang D., Chen Y., Cao T., Wang J., Lin C. (2024). A cross-linked macropore hydrogel based on M1 macrophage lysate and alginate regulates tumor-associated macrophages for the treatment of melanoma. Int. J. Biol. Macromol..

[72] Wu Q., Jia K., Liu Y., Zhang Y., Jiang N., Li L., Liu Y., Li L. (2026). The effect of the physicochemical properties of hydrogels on chronic inflammation by macrophage polarization. Biomater. Sci..

[73] Dong H., Li Q., Zhang Y., Ding M., Teng Z., Mou Y. (2023). Biomaterials Facilitating Dendritic Cell-Mediated Cancer Immunotherapy. Adv. Sci..

[74] Steinman R.M., Banchereau J. (2007). Taking dendritic cells into medicine. Nature.

[75] Chang H., Wen X., Li Z., Ling Z., Zheng Y., Xu C. (2023). Co-delivery of dendritic cell vaccine and anti-PD-1 antibody with cryomicroneedles for combinational immunotherapy. Bioeng. Transl. Med..

[76] Liu X.Y., Zhuang Y.P., Huang W., Wu Z.Z., Chen Y.J., Shan Q.A., Zhang Y.F., Wu Z.Y., Ding X.Y., Qiu Z.L. (2023). Interventional hydrogel microsphere vaccine as an immune amplifier for activated antitumour immunity after ablation therapy. Nat. Commun..

[77] Yoshizaki Y., Horii K., Murase N., Kuzuya A., Ohya Y. (2024). Development of immune cell delivery system using biodegradable injectable polymers for cancer immunotherapy. Int. J. Pharm..

[78] Friedl P., Brocker E.B. (2000). T cell migration in three-dimensional extracellular matrix: Guidance by polarity and sensations. Dev. Immunol..

[79] Lammermann T., Sixt M. (2009). Mechanical modes of ‘amoeboid’ cell migration. Curr. Opin. Cell Biol..

[80] Jacobelli J., Friedman R.S., Conti M.A., Lennon-Dumenil A.M., Piel M., Sorensen C.M., Adelstein R.S., Krummel M.F. (2010). Confinement-optimized three-dimensional T cell amoeboid motility is modulated via myosin IIA-regulated adhesions. Nat. Immunol..

[81] Lammermann T., Bader B.L., Monkley S.J., Worbs T., Wedlich-Soldner R., Hirsch K., Keller M., Forster R., Critchley D.R., Fassler R. (2008). Rapid leukocyte migration by integrin-independent flowing and squeezing. Nature.

[82] Wang W., Liu Y., He Z., Li L., Liu S., Jiang M., Zhao B., Deng M., Wang W., Mi X. (2024). Breakthrough of solid tumor treatment: CAR-NK immunotherapy. Cell Death Discov..

[83] Balkhi S., Zuccolotto G., Di Spirito A., Rosato A., Mortara L. (2025). CAR-NK cell therapy: Promise and challenges in solid tumors. Front. Immunol..

[84] Shimasaki N., Jain A., Campana D. (2020). NK cells for cancer immunotherapy. Nat. Rev. Drug Discov..

[85] Minopoli A., Perini G., Cui L., Palmieri V., De Spirito M., Papi M. (2025). Biomaterial-driven 3D scaffolds for immune cell expansion toward personalized immunotherapy. Acta Biomater..

[86] Felices M., Chu S., Kodal B., Bendzick L., Ryan C., Lenvik A.J., Boylan K.L.M., Wong H.C., Skubitz A.P.N., Miller J.S. (2017). IL-15 super-agonist (ALT-803) enhances natural killer (NK) cell function against ovarian cancer. Gynecol. Oncol..

[87] Luo J., Guo M., Huang M., Liu Y., Qian Y., Liu Q., Cao X. (2025). Neoleukin-2/15-armored CAR-NK cells sustain superior therapeutic efficacy in solid tumors via c-Myc/NRF1 activation. Signal Transduct. Target. Ther..

[88] Baughan S.L., Folsom T., Johnson M., Kreuger J., Webber B., Moriarity B.S. (2025). Perspective: IL-15 cytokine-armored NK cells as ready-to-use immunotherapy for diverse malignancies: Therapeutic potential and toxicity risks. Front. Immunol..

[89] Johnson M., Lloyd J., Tekkam S., Crooke S.N., Witherden D.A., Havran W.L., Finn M.G. (2020). Degradable Hydrogels for the Delivery of Immune-modulatory Proteins in the Wound Environment. ACS Appl. Bio Mater..

[90] Pi Y.P., Ganabady K., Celiz A.D. (2025). Enzyme-responsive biomaterials for biomedical applications. Commun. Mater..

[91] Hoffmann J.C., Schön M.P. (2021). Integrin αE (CD103) β7 in epithelial cancer. Cancers.

[92] Chen S., Saeed A., Liu Q., Jiang Q., Xu H., Xiao G.G., Rao L., Duo Y. (2023). Macrophages in immunoregulation and therapeutics. Signal Transduct. Target. Ther..

[93] Blank C.U., Haining W.N., Held W., Hogan P.G., Kallies A., Lugli E., Lynn R.C., Philip M., Rao A., Restifo N.P. (2019). Defining ‘T cell exhaustion’. Nat. Rev. Immunol..

[94] Guo R., Wang R., Zhang W., Li Y., Wang Y., Wang H., Li X., Song J. (2025). Macrophage Polarisation in the Tumour Microenvironment: Recent Research Advances and Therapeutic Potential of Different Macrophage Reprogramming. Cancer Control.

[95] Zhao Y., Ni Q., Zhang W., Yu S. (2024). Progress in reeducating tumor-associated macrophages in tumor microenvironment. Discov. Oncol..

[96] Solanki R., Bhatia D. (2024). Stimulus-Responsive Hydrogels for Targeted Cancer Therapy. Gels.

[97] Han B., Song Y., Park J., Doh J. (2022). Nanomaterials to improve cancer immunotherapy based on ex vivo engineered T cells and NK cells. J. Control Release.

[98] Yilmaz A.S., Pepe A., Latifi N., Sawyer G.W., Dunne M.R. (2026). Stimuli-responsive hydrogels in targeted cancer therapy: A systematic review of materials, mechanisms, and delivery strategies. Mater. Des..

[99] Stephan S.B., Taber A.M., Jileaeva I., Pegues E.P., Sentman C.L., Stephan M.T. (2015). Biopolymer implants enhance the efficacy of adoptive T-cell therapy. Nat. Biotechnol..

[100] Smith T.T., Moffett H.F., Stephan S.B., Opel C.F., Dumigan A.G., Jiang X.Y., Pillarisetty V.G., Pillai S.P.S., Wittrup K.D., Stephan M.T. (2017). Biopolymers codelivering engineered T cells and STING agonists can eliminate heterogeneous tumors. J. Clin. Investig..

[101] Zeng Y.C., Sun Y.Q., Zhang X.Q., Liang S.J., Chen L.L., Zhang K.Y., Lu Z.D., Lai P. (2025). Injectable tert-butylphenylacetic acid/acrylated beta-cyclodextrin-based hydrogels for co-delivery of CAR-T cells and IL-15 in solid tumor therapy. Mater. Today Bio.

[102] Nam S., Mooney D. (2021). Polymeric Tissue Adhesives. Chem. Rev..

[103] Xu K.L., Di Caprio N., Fallahi H., Dehghany M., Davidson M.D., Laforest L., Cheung B.C.H., Zhang Y., Wu M., Shenoy V. (2024). Microinterfaces in biopolymer-based bicontinuous hydrogels guide rapid 3D cell migration. Nat. Commun..

[104] Rijns L., Rutten M., Vrehen A.F., Aldana A.A., Baker M.B., Dankers P.Y.W. (2024). Mimicking the extracellular world: From natural to fully synthetic matrices utilizing supramolecular biomaterials. Nanoscale.

[105] Rezaei Z., Yilmaz-Aykut D., Tourk F.M., Bassous N., Barroso-Zuppa M., Shawl A.I., Ashraf S.S., Avci H., Hassan S. (2022). Immunomodulating Hydrogels as Stealth Platform for Drug Delivery Applications. Pharmaceutics.

[106] Zeng J., Jin Y., Ao Y., Li W., Liang K., Chen H., Long Y., Du Y. (2024). Modulating immune responses for enhanced cell therapies: The dual role of multi-scale biomaterials. EngMedicine.

[107] Joyce J.A., Fearon D.T. (2015). T cell exclusion, immune privilege, and the tumor microenvironment. Science.

[108] Huang S., Zhao Y., Shen J. (2025). Advances in integrating biomaterials with CAR-T cells for enhancing solid tumor therapy. Chin. J. Cancer Res..

[109] Correia D., Faneca H., Wang H.L., Cordeiro R. (2026). Hydrogels as delivery platforms for advancing T cell therapies. J. Drug Deliv. Sci. Technol..

[110] Jorgensen L.V., Christensen E.B., Barnkob M.B., Barington T. (2025). The clinical landscape of CAR NK cells. Exp. Hematol. Oncol..

[111] Vahidi S., Zabeti Touchaei A., Samadani A.A. (2024). IL-15 as a key regulator in NK cell-mediated immunotherapy for cancer: From bench to bedside. Int. Immunopharmacol..

[112] Kim H. (2025). Overcoming Immune Barriers in Allogeneic CAR-NK Therapy: From Multiplex Gene Editing to AI-Driven Precision Design. Biomolecules.

[113] Martin K.E., Hammer Q., Perica K., Sadelain M., Malmberg K.J. (2024). Engineering immune-evasive allogeneic cellular immunotherapies. Nat. Rev. Immunol..

[114] Myers J.A., Miller J.S. (2021). Exploring the NK cell platform for cancer immunotherapy. Nat. Rev. Clin. Oncol..

[115] Casaramona A.S., Bachmann M.F., Sevick-Muraca E., Mohsen M.O. (2026). γδ T cells at the interface of innate and adaptive immunity in cancer. J. Immunother. Cancer.

[116] Stevens G., Blanco-Dominguez R., Mensurado S., Silva-Santos B. (2026). gammadelta T Cells for Cancer Immunotherapy: Unconventional Players Take the Spotlight. Annu. Rev. Immunol..

[117] Fang X., Yan S., He L., Deng C. (2025). CAR-gammadelta T cells: A new paradigm of programmable innate immune sentinels and their systemic applications in cancer and beyond. Front. Immunol..

[118] Silva-Santos B., Serre K., Norell H. (2015). γδ T cells in cancer. Nat. Rev. Immunol..

[119] Schenkel J.M., Pauken K.E. (2023). Localization, tissue biology and T cell state-implications for cancer immunotherapy. Nat. Rev. Immunol..

[120] Mensurado S., Blanco-Dominguez R., Silva-Santos B. (2023). The emerging roles of γδ T cells in cancer immunotherapy. Nat. Rev. Clin. Oncol..

[121] Wang C.Q., Lim P.Y., Tan A.H. (2023). Gamma/delta T cells as cellular vehicles for anti-tumor immunity. Front. Immunol..

[122] Luo X., Lv Y., Yang J., Long R., Qiu J., Deng Y., Tang G., Zhang C., Li J., Zuo J. (2024). Gamma delta T cells in cancer therapy: From tumor recognition to novel treatments. Front. Med..

[123] Block A., Liu X., Zhang D., Adebowale K. (2026). Engineering macrophages for cancer immunotherapy: Emerging insights and therapeutic potential. npj Biomed. Innov..

[124] Bencherif S.A., Warren Sands R., Ali O.A., Li W.A., Lewin S.A., Braschler T.M., Shih T.Y., Verbeke C.S., Bhatta D., Dranoff G. (2015). Injectable cryogel-based whole-cell cancer vaccines. Nat. Commun..

[125] Leach D.G., Dharmaraj N., Piotrowski S.L., Lopez-Silva T.L., Lei Y.L., Sikora A.G., Young S., Hartgerink J.D. (2018). STINGel: Controlled release of a cyclic dinucleotide for enhanced cancer immunotherapy. Biomaterials.

[126] Ali O.A., Emerich D., Dranoff G., Mooney D.J. (2009). In situ regulation of DC subsets and T cells mediates tumor regression in mice. Sci. Transl. Med..

[127] Irvine D.J., Hanson M.C., Rakhra K., Tokatlian T. (2015). Synthetic Nanoparticles for Vaccines and Immunotherapy. Chem. Rev..

[128] Riley R.S., June C.H., Langer R., Mitchell M.J. (2019). Delivery technologies for cancer immunotherapy. Nat. Rev. Drug Discov..

[129] Bleriot C., Chakarov S., Ginhoux F. (2020). Determinants of Resident Tissue Macrophage Identity and Function. Immunity.

[130] Ginhoux F., Guilliams M. (2016). Tissue-Resident Macrophage Ontogeny and Homeostasis. Immunity.

[131] Previtera M.L., Sengupta A. (2015). Substrate Stiffness Regulates Proinflammatory Mediator Production through TLR4 Activity in Macrophages. PLoS ONE.

[132] Meli V.S., Atcha H., Veerasubramanian P.K., Nagalla R.R., Luu T.U., Chen E.Y., Guerrero-Juarez C.F., Yamaga K., Pandori W., Hsieh J.Y. (2020). YAP-mediated mechanotransduction tunes the macrophage inflammatory response. Sci. Adv..

[133] Atcha H., Jairaman A., Holt J.R., Meli V.S., Nagalla R.R., Veerasubramanian P.K., Brumm K.T., Lim H.E., Othy S., Cahalan M.D. (2021). Mechanically activated ion channel Piezo1 modulates macrophage polarization and stiffness sensing. Nat. Commun..

[134] Jain N., Vogel V. (2018). Spatial confinement downsizes the inflammatory response of macrophages. Nat. Mater..

[135] McWhorter F.Y., Wang T., Nguyen P., Chung T., Liu W.F. (2013). Modulation of macrophage phenotype by cell shape. Proc. Natl. Acad. Sci. USA.

[136] Ghasemiyeh P., Mohammadi-Samani S. (2019). Hydrogels as drug delivery systems; pros and cons. Trends Pharm. Sci. Technol..

[137] Cristiana S.A.B., Gaspar M.C., Coimbra P., de Sousa H.C., Mara E.M.B. (2023). A review of conventional and emerging technologies for hydrogels sterilization. Int. J. Pharm..

[138] Fowler A., Toner M. (2006). Cryo-injury and biopreservation. Ann. N. Y. Acad. Sci..

[139] Egorikhina M.N., Rubtsova Y.P., Linkova D.D., Charykova I.N., Farafontova E.A., Aleinik D.Y. (2024). Specifics of Cryopreservation of Hydrogel Biopolymer Scaffolds with Encapsulated Mesenchymal Stem Cells. Polymers.

[140] Chaudhuri O. (2017). Viscoelastic hydrogels for 3D cell culture. Biomater. Sci..

[141] Bulte J.W.M., Daldrup-Link H.E. (2018). Clinical Tracking of Cell Transfer and Cell Transplantation: Trials and Tribulations. Radiology.

[142] Sebestyen Z., Prinz I., Dechanet-Merville J., Silva-Santos B., Kuball J. (2020). Translating gammadelta (gammadelta) T cells and their receptors into cancer cell therapies. Nat. Rev. Drug Discov..

[143] Silva-Santos B., Mensurado S., Coffelt S.B. (2019). gammadelta T cells: Pleiotropic immune effectors with therapeutic potential in cancer. Nat. Rev. Cancer.

